# Prolonged Oral Administration of Oleuropein Might Protect Heart against Aconitine-induced Arrhythmia 

**Published:** 2012

**Authors:** Mansour Esmailidehaj, Seyed-Jalil Mirhosseini, Mohammad Ebrahim Rezvani, Bahram Rasoulian, Mohammad Hossein Mosaddeghmehrjardi, Damoon Haghshenas

**Affiliations:** a *Department of Physiology, Faculty of Medicine, Shahid Sadoughi University of Medical Sciences, Yazd, Iran. *; b *Cardiovascular Research Center, Afshar Hospital, Shahid Sadoughi University of Medical Sciences, Yazd, Iran. *; c *Razi Herbal Medicines Research Center, Lorestan University of Medical sciences, Khorramabad, Iran. *; d *Department of Pharmacology, Faculty of Medicine, Shahid Sadoughi University of Medical Sciences, Yazd, Iran. *

**Keywords:** Oleuropein, Aconitine, Rat, Ventricular fibrillation, Ventricular tachycardia, Arrhythmia

## Abstract

In this study, it was surveyed to know whether an oral single dose of oleuropein could mimic the cardiac preconditioning in rats’ hearts or whether its prolonged oral administration could protect the heart against the aconitine-induced arrhythmia in rats.

Eighty male Wistar rats were divided into two series (n = 8 in each group). In the first series, all groups (except the control (Con) group) were given a single oral dose of oleuropein (20 mg/Kg) 1, 3, 24 and 48 h before the infusion of aconitine. In the second series, except the Con group, the other four groups were given oral oleuropein (20 mg/Kg/day) for 3, 7, 14 and 28 days, before the infusion of aconitine. Electrocardiogram was recorded to monitor arrhythmia.

Data of the first series showed that the initiation time of arrhythmia, the initiation of ventricular tachycardia (VT), the numbers of reversible ventricular fibrillation (VF) and the death time had no significant difference compared with Con group. In the second series, a significant protection was occurred only in the 28 days group that was evident with increased initiation time of arrhythmia, increased initiation time of VT, and increased the number of reversible VF and death time in compared to the Con group.

The findings of this study show that the oral administration of a single dose of oleuropein could not mimic the preconditioning effects in rat hearts, but the prolonged administration of oleuropein for about four weeks could protect the heart against aconitine-induced arrhythmia.

## Introduction

Nowadays, cardiac arrhythmias have attracted a great attention in cardiovascular research around the world. Life-threatening arrhythmia including ventricular tachycardia (VT) and ventricular fibrillation (VF) are accounted for many cases of cardiac deaths, especially after the coronary artery bypass graft (CABG) (1, 2). Although the precise mechanism of arrhythmia is unknown, it has been documented that hemodynamic instability, transient metabolic disorders and anatomical electrical substrates play an important role in the incidence of arrhythmia following CABG (2). On the other hand, the most common antiarrhythmic drugs such as lidocaine increase the cardiac vulnerability through their proarrhythmic effects (3). So, finding new safe compounds, especially natural compounds, might have an important role in decreasing the incidence of arrhythmia and enhancing the survival rate.

Oleuropein is one of the natural compounds that have been investigated in many studies. Oleuropein is a phenolic compound that is found in large quantities in olive leaves (4, 5). It constitutes about 6-9% of the dry matter of olive leaves (6, 7). Several recent studies have shown that oleuropein has antioxidant (5, 8), hypoglycemic and antidiabetic (9, 10), hypolipidemic and anti-atherosclerotic (11), hypotensive (4, 5), antiapoptic (12), antimicrobial (8) and anti-inflammatory (5, 13) effects.

Most studies believed that these bioactive effects of oleuropein are related to its antioxidant property (5, 9, 10, 14). The higher oleuropein concentration in olive leaf extract causes the greater antioxidant property (15). On the other hand, some studies have shown that oleuropein could modify the intracellular pathways and it even produces free radicals (9). Recently, our study showed that pre-exposure to brief episodes of oxygen radicals (with exposure to hyperoxia) could precondition rat hearts against the ischemic injury (16, 17). Preconditioning is a phenomenon thereby a sublethal ischemia or pharmacological agent protects the heart against the subsequent prolonged ischemia through a biphasic mode (18): the early phase immediately starts following the insertion of stimulus and lasts for 1-3 h and the delayed phase initiates about 24 h later and continue up to 72 h (16).

For that reason, the main goal of this study was that whether a single dose of oleuropein could mimic both phases of preconditioning against aconitine-induced arrhythmia. If not, whether its prolonged administration for several days could protect the heart in opposition to the aconitine-induced arrhythmia?

## Experimental

*Animals*

Male Wistar rats, weighing 250-300 g, were used to perform this study. All animals were housed under the standard conditions with 12 h dark/light cycle, humidity of 50% and temperature of 22 ± 2°C. All animals had freely access to food and water ad libitum. This study was conducted in accordance with the guides for the Care and Use of Animal Laboratory of Shahid Sadoughi University of Medical Sciences, Iran.

*Experimental groups*

Eighty rats were divided into two series of five groups (n = 8). In the first series, to determine whether oleuropein has preconditioning effect, the control (Con) group was received no treatment and the other four groups received a single oral dose of 20 mg/Kg oleuropein (Extrasynthese, France), 1, 3, 24 and 48 h before the intravenous infusion of 0.2 μg/min aconitine (Sigma, USA), respectively (Ole1, Ole3, Ole24 and Ole48 groups). The infusion of aconitine has been continued till the heart shows irreversible VF or animal death.

Since in the first series, the preconditioning effect was not observed by single dose of oleuropein, in the second series, oleuropein was given to rats for several days to know the cardioprotective effect. The Con group was given no treatment and the other four groups were given 20 mg/Kg/day oleuropein for 3, 7, 14 and 28 days (O3, O7, O14 and O28 groups), respectively. Then, 0.2 μg/min aconitine was infused in their tail vein till the heart shows irreversible VF or animal death. The dose of oleuropein was selected according to the previous work by Andreadou *et al. *(11).

*Surgical procedure*

All rats were anaesthetized with intraperitoneal sodium thiopental (100 mg/Kg). Then, their tail vein was cannulated with an angiocatheter (Gauge 23) to infuse aconitine (0.2 μg/min) and carotid artery cannulated to measure the direct blood pressure. Next, Body temperature was kept around 37°C by a heat pad. Afterward, to monitor the electrocardiogram (ECG), limb lead II was recorded by attaching negative and positive electrodes to the right hand and the left foot, respectively. Finally, all data including blood pressure, heart rate and ECG were saved on computer using power lab data acquisition apparatus (Lab Chart 7, ADI, Australia). *Arrhythmia assessment*

Following 20 min of stabilization, to induce arrhythmia, aconitine was infused intravenously (0.2 μg/min) by an infusion pump till the rat died from arrhythmia. These type of arrhythmia were started about four min after the continuous infusion of aconitine in the Con group. Arrhythmias were analyzed according to lambeth conventions (19): Ventricular ectopic beats (VEBs) were defined as one to three wide QRS complexes that lacked of P wave. If there was a run of four or more consequent VEBs, it was considered as VT and when there was no distinguishable wave and blood pressure was decreased significantly, it was defined as VF. Finally, if VF was continued for more than 120 min, it was considered as irreversible VF and death.

*Statistics*

Data were shown as mean ± SEM. Since the data had no normal distribution, they were analyzed with non-parametric Kruskal-Wallis test. p < 0.05 was considered statistically significant.

## Results

*First series*

*Hemodynamic parameters*

**Figure 1:**
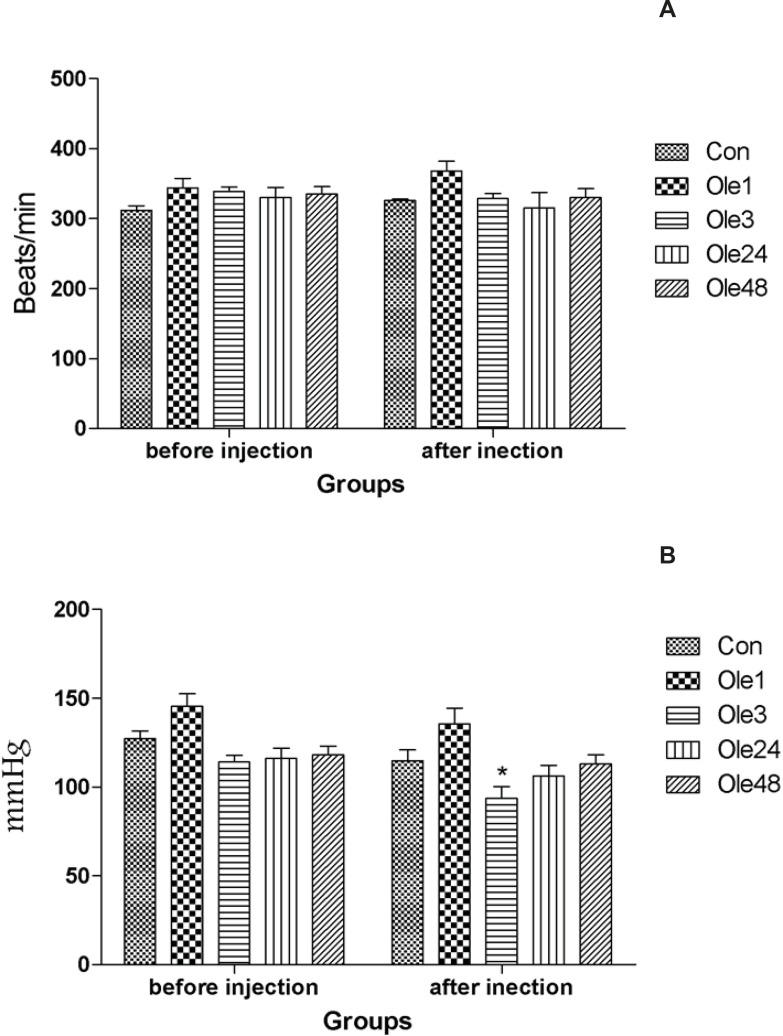
Hemodynamic parameters of rats received an oral single dose of 20 mg/Kg oleuropein before the intravenous infusion of aconitine (0.2 μg/min). A: Heart rate; B: Mean blood pressure. Con: control group; Ole1, Ole3, Ole24 and Ole48: mean groups that were given a single dose of oleuropein, 1, 3, 24 and 48 h before the infusion of aconitine, respectively. *: p < 0.05

The heart rate was almost similar in all groups at the baseline and before the beginning of arrhythmia (Figure 1A). Besides, the mean arterial blood pressure was almost similar at the baseline in all groups. However, it was decreased before the beginning of arrhythmia in all groups that was only significant in Ole3 group compared to the Con group (Figure 1B).

*Arrhythmia*

**Figure 2:**
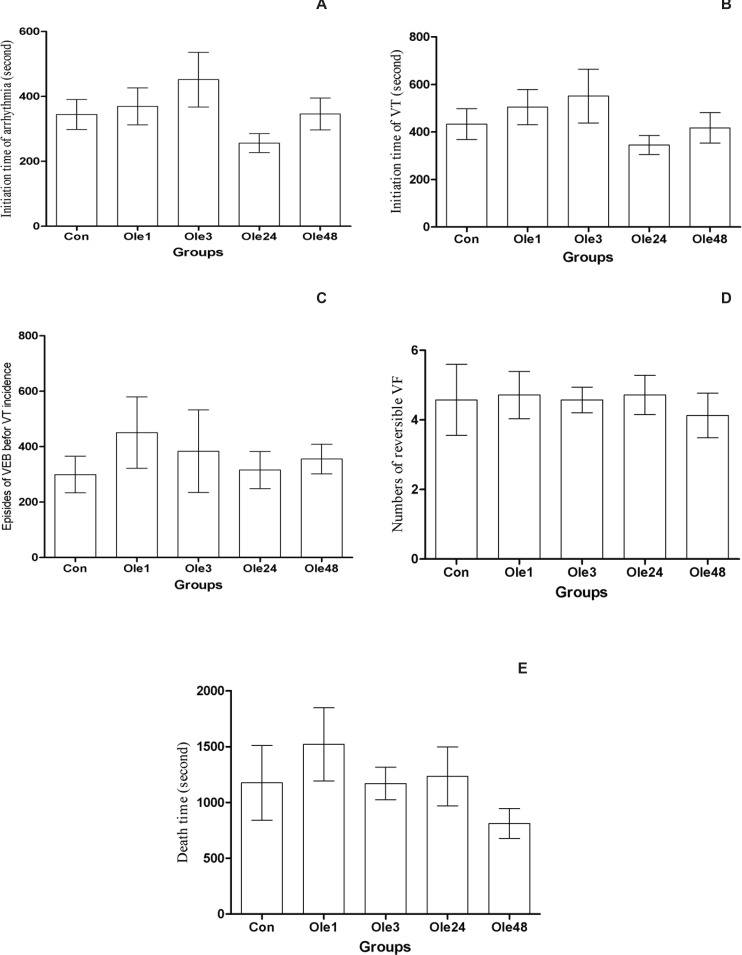
Effect of an oral single dose of oleuropein (20 mg/Kg) on preconditioning against the aconitine-induced arrhythmia in rats A: The initiation time of arrhythmia; B: The initiation time of ventricular tachycardia (VT); C: The episodes of ventricular ectopic beats before VT incidence; D: The number of reversible ventricular fibrillation (VF) and E: The death time. Con: control group; Ole1, Ole3, Ole24 and Ole48: mean groups that were given oleuropein 1, 3, 24 and 48 h before the infusion of aconitine, respectively. *: p < 0.05

The mean initiation time of arrhythmia (always occurred as VEBs) was not significantly increased in all groups (Figure 2A).

The initiation mean time of VT following the administration of aconitine had not any significant increase compared with the Con group (Figure 2B).

Although the episodes of VEBs before the incidence of VT were reduced in Ole24 and Ole48, there was no significant difference between these two groups (Figure 2C).

Increase of reversible VF number during continuous infusion of aconitine is an indicator of the increased resistant of heart to complete arrest. In the first series, the average number of reversible VF did not have any significant difference among all groups (Figure 2D).

*Second series*

*Hemodynamic parameters*

**Figure 3:**
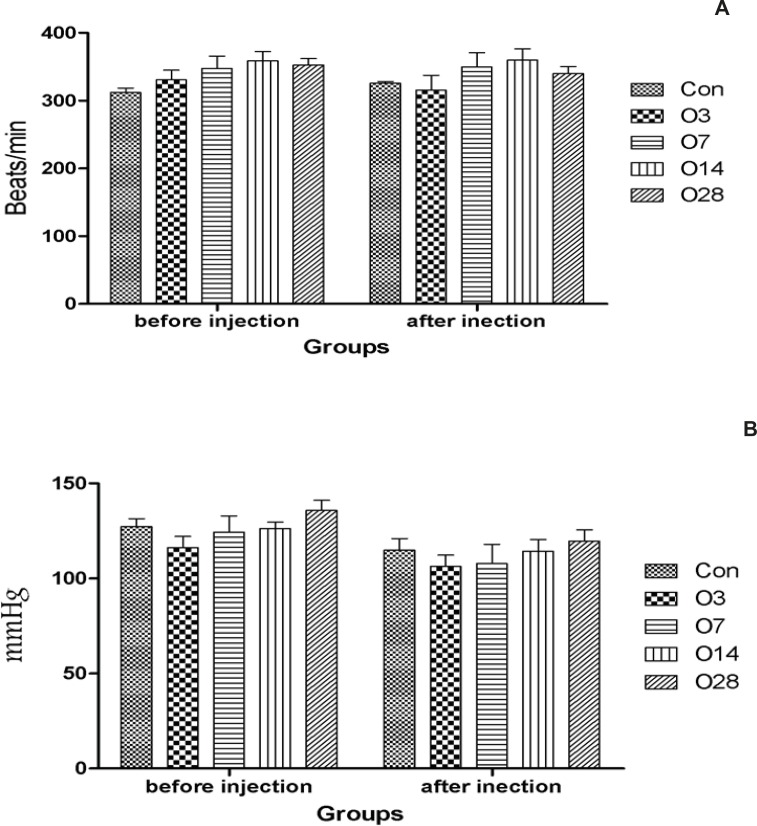
Hemodynamic parameters of rats received 20 mg/Kg oleuropein for several days before the intravenous infusion of aconitine. A: Heart rate; B: Mean blood pressure; Con: control group; O3, O7, O14 and O28: mean groups that were received oleuropein for 3, 7, 14 and 28 days before the infusion of aconitine (0.2 μg/min), respectively. *: p < 0.05 vs Con group

Although after the infusion of aconitine, the mean arterial pressure has been reduced in all groups, there was no significant difference in heart rate and mean blood pressure before and after the infusion of aconitine among all groups (Figure 3).

*Arrhythmia*

**Figure 4:**
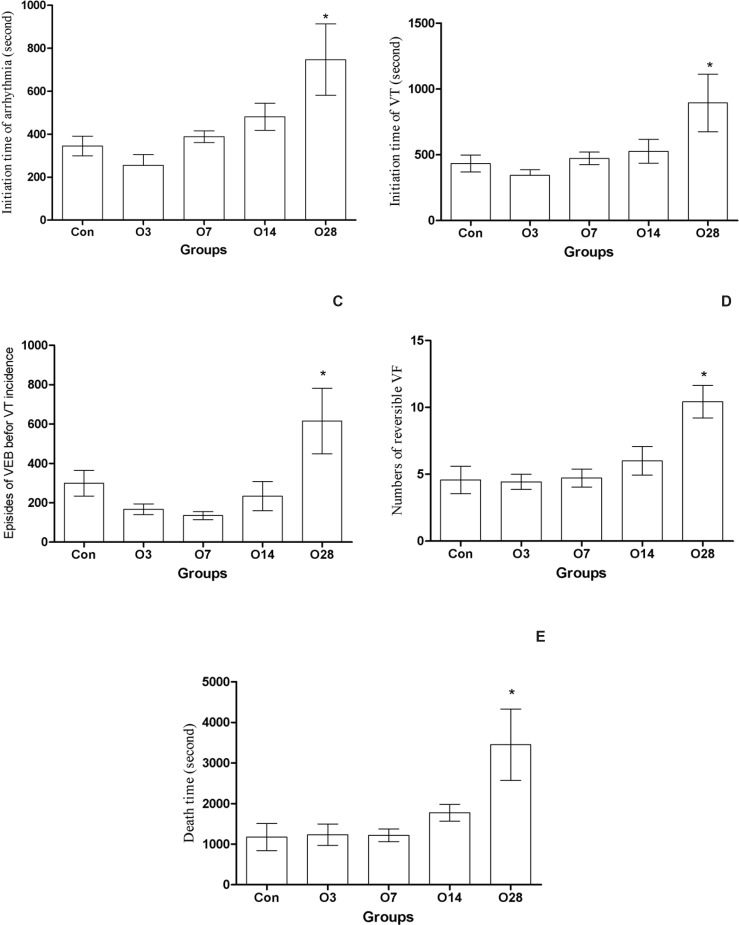
Effect of oral administration of oleuropein (20 mg/Kg) for several days against aconitine-induced arrhythmia in rats. A: The initiation time of arrhythmia; B: The initiation time of ventricular tachycardia (VT); C: The episodes of ventricular ectopic beats before VT incidence; D: The number of reversible ventricular fibrillation (VF); E: The death time. Con: control group; O3, O7, O14 and O28: mean groups that received oleuropein for 3, 7, 14 and 28 days before the infusion of aconitine (0.2 μg/min), respectively. *: p < 0.05 vs Con group

The mean initiation time of arrhythmia has only been increased significantly in the O28 group, 675 ± 157, compared with the Con group, 344 ± 45, (Figure 4A).

The mean initiation time of VT after the infusion of aconitine was increased significantly in the O28 group, 894 ± 218, compared with the Con group, and 433 ± 65, (Figure 4B).

The mean episode of VEBs between the initiation time of arrhythmia and VT was only increased in the O28 group, 515 ± 166, in comparison with the Con group, 299 ± 65, (Figure 4C).

The number of reversible VF that is an indicator of increased resistant of heart against the complete arrest was only remarkably increased in the O28 group (10.4 ± 1.2), compared with the Con group (4.1 ± 1) (Figure 4D).

Furthermore, the mean death time was only increased in the O28 group (3451 ± 880), compared with the Con group (1176 ± 335) (Figure 4E).

## Discussion

The findings of this study show that a single oral dose of oleuropein (20 mg/Kg/day) does not have preconditioning-like effect against aconitine-induced arrhythmia in rat hearts, however, its prolonged oral administration, especially for 28 days, could protect the heart against the aconitine-induced arrhythmia that was evident with increased initiation time of arrhythmia and increasing the number of reversible VF and death time.

Increasing the antioxidant capacity of body may lead to decrease in the incidence of arrhythmia. Oleuropein is a natural powerful antioxidant compound that has many beneficial effects in animals and human beings (5, 14, 20, 21). It is believed that the high resistant of olive tree to pathogens and insect attacks are related to its high phenolic antioxidant compounds, especially oleuropein (22).

Though, most biological benefits of oleuropein are attributed to its antioxidant activity. Some investigations have shown that oleuropein has also non-antioxidant activity. For instance, OI-Kano and his colleagues in 2008 shown that oleuropein-rich diet (0.1-0.4%) increases the secretion of catecholamines into the rat blood (23). Mora Sonticogo *et al. *in 2010 reported that exposing the rat mesanchymal stem cells for 7-21 days could increase their differentiation to osteoblasts and decrease to adipocytes and osteoclasts (24). Other study has shown that oleuropein increases the production of nitric oxide in mouse macrophage and antagonizes calcium channels (25).

One of the goals of our study was that whether the oral administration of a single dose of oleuropein (the dose that has been used by most previous studies) has preconditioning-like effects against the aconitine-induced arrhythmia. Preconditioning is a phenomenon whereby exposing an organ to brief sublethal stimuli (ischemia, pharmacological or mechanical stimulus) increases the tolerance of that organ in opposition to lethal stresses (2, 26).

In our previous study, it was shown that inducing a transient low systemic oxidative stress through exposing the rats to hyperoxic environment could elicit a delayed preconditioning effect against the ischemic injury (16). Since oleuropein has dual effects on oxidative stress (9), namely increases the production of intracellular free radicals in one hand, and scavenges the free radicals by its hydroxyl group on the other hand, it might precondition the heart against aconitine-induced arrhythmia through its oxidative effect. But our results showed that this dose does not have a preconditioning effect (Figure 2A-E). In another study of ours, a single dose of oleuropein (100 mg/Kg) was first injected and then, the rat hearts were subjected to ischemic-reperfusion injury after 1, 3, 6, 24 and 48 h under langendorff apparatus. Oleuropein had not preconditioning effects against the ischemic injury, but it had a cardioprotective effect up to 3 h that may be related to its antioxidant activity (unpublished data).

Previous studies have reported that oleuropein is rapidly absorbed from the intestine and reaches to its maximum absorption 2 h later. Then, it is distributed throughout the body (12, 23, 27). For this reason, to evaluate the preconditioning effect of oleuropein, it was applied 2 h to each group till the oleuropein had sufficient time to be absorbed from intestine. To our knowledge, there is no study about oleuropein and preconditioning. However, two studies have reported the cardioprotective effect of a single dose of oleuropein (28, 29). Petkove and Manolov in 1978 reported that intravenous administration of oleuropein (40 mg/Kg, once) has antiarrhythmic effects (29). Manna *et al. *in 2004 addressed that perfusing the isolated rat hearts with oleuropein for 15 min could reduce the reperfusion oxidative stress (28).

Since in first series we did not observe preconditioning effects against aconitine-induced arrhythmia with a single dose of oleuropein, the animals were treated with the same dose of oleuropein for different times (3, 7, 14 and 28 days) to know whether increasing the duration of treatment could mimic the cardioprotective effects. As the results showed (Figure 4), increasing the duration of pretreatment to 4 weeks could induce protection against the aconitine-induced arrhythmia. This protection was evidence with the increased initiation time of arrhythmia, reversible VF and animal death time. Since the heart rate and blood pressure had not any significant different between the groups, these effects are not dependent on hemodynamic parameters. Further studies need to determine whether higher doses of oleuropein (orally) could reduce this duration of treatment.

In some other studies, the duration of treatment with oleuropein has influenced its effects. For instance, Sonticogo Mora *et al. *in 2010 reported that the effect of oleuropein on differentiation of mesanchymal stem cells to osteoblasts is time-dependent. In this study, the treatment of cells with oleuropein for 14 and 21 days had better effects comparing with 7 days (24). Ioanna Andreadou *et al. *reported that the feeding of normolipidemic and hyperlipidemic rabbits with oleuropein (10 and 20 mg/Kg/day) for 3 and 6 weeks had an anti-infarct effect (11). However, they did not point to anything arrhythmia. In another study, they reported that intraperitoneal administration of oleuropein (100 and 200 mg/Kg/day for 5 days) could reduce the cardiotoxicity effect of a single dose of intraperitoneal doxorubicin (20 mg/Kg) in rats (30, 31).

In summary, an oral single dose of oleuropein could not precondition the rat hearts against the aconitine-induced arrhythmia and further studies need to investigate the higher doses. On the other hand, by increasing the duration of treatment to four weeks, oleuropein could protect the heart against the aconitine-induced arrhythmia and still further studies are needed to test the effect of higher doses of oleuropein on the duration of treatment with oleuropein.
